# Lipid Accumulation Product and Cardiometabolic Index as Effective Tools for the Identification of Athletes at Risk for Metabolic Syndrome

**DOI:** 10.3390/life14111452

**Published:** 2024-11-08

**Authors:** Giuseppe Di Gioia, Armando Ferrera, Mihail Celeski, Raffaella Mistrulli, Erika Lemme, Federica Mango, Maria Rosaria Squeo, Antonio Pelliccia

**Affiliations:** 1 Institute of Medicine and Sport Science, National Italian Olympic Committee, Largo Piero Gabrielli, 1, 00197 Rome, Italy; dottgiuseppedigioia@gmail.com (G.D.G.); armando.ferrera95@gmail.com (A.F.); erikalemme@msn.com (E.L.); federicamango.md@gmail.com (F.M.); mariarosaria.squeo@coni.it (M.R.S.); 2 Department of Movement, Human and Health Sciences, University of Rome “Foro Italico”, Piazza Lauro de Bosis, 15, 00135 Rome, Italy; 3 Clinical and Molecular Medicine Department, Sapienza University of Rome, 00198 Rome, Italy; raffaella.mistrulli@uniroma1.it; 4 Department of Cardiovascular Sciences, Fondazione Policlinico Universitario Campus Bio-Medico, Via Alvaro del Portillo, 200, 00128 Rome, Italy; mihail.celeski@unicampus.it

**Keywords:** metabolic syndrome, athletes, Olympics, lipid accumulation product, cardiometabolic index, cardiovascular disease

## Abstract

Introduction: Metabolic syndrome (MS) is a growing global public health concern that is associated with increased risk for cardiovascular events, even in athletes. The lipid accumulation product (LAP) index and cardiometabolic index (CMI) have been shown to be efficient markers of MS in the general population; its applicability in athletes has not been discussed yet. We aimed to assess the role of LAP and CMI in predicting MS in athletes. Methods: We retrospectively enrolled 793 Olympic athletes practicing different sporting disciplines (power, skill, endurance, and mixed), classified arbitrarily into no risk (NR), low risk (LR), high risk (HR), or MS if they had 0, 1, 2, or 3 criteria for MS, respectively. Evaluations included a calculation of the LAP index, CMI, anthropometric measurements, and clinical and laboratorial variables. Results: Among our population, only 0.8% reached the criteria for MS, 9.1% were at HR for MS, 37.8% were defined as LR, and 52.3% had NR. Significant differences in anthropometric parameters and the principal components of MS criteria (blood pressure, lipidic profile, glycemia) were reported predominantly in HR athletes and those with MS (*p* < 0.0001). LAP and CMI presented linearly increasing values from individuals with NR to those with MS (*p* < 0.0001). In addition, HR and MS athletes were classified as “likely MS” (9.8%) and LR and NR athletes as “unlikely MS” (90.2%). After adjusting for potential confounders, LAP ≥ 34.66 and CMI ≥ 0.776 emerged as independent predictors for MS in the overall cohort (Hazar Ratio (HR) 7.22 [3.75–13.89], *p* < 0.0001, and HR 5.37 [2.96–9.73], *p* < 0.0001, respectively). The ROC curve revealed that these cut-offs in the general population predict MS with an area under the curve (AUC) of 0.80 and 0.79, respectively, for LAP and CMI. However, gender-related cut-offs seem to be more precise in predicting MS (LAP ≥ 38.79 for male, LAP ≥ 14.16 for female, and CMI ≥ 0.881 for male and ≥0.965 for female). Conclusion: The ROC curve analyses of LAP and CMI showed good diagnostic accuracy in predicting MS among athletes, despite the low prevalence of MS in our sample. Thus, these indexes may be used to promote screening for primary prevention and early detection of athletes at risk for MS to establish an early prevention strategy. Larger prospective studies are necessary to validate their benefit in the general population.

## 1. Introduction

Metabolic syndrome (MS) is a rising worldwide health issue. It is a pluri-pathological state defined by the co-occurrence of multiple cardiovascular risk factors, including high blood pressure, insulin resistance, abdominal obesity, and lipid metabolism disorders (high triglycerides, low high-density lipoprotein (HDL)-cholesterol) [1]. Additionally, it is linked to higher rates of morbidity, death, and healthcare costs [2,3].

There are numerous MS prediction models that have been put forward. Research has shown that variables such as age, sex, γ-glutamyl transpeptidase (GGT), uric acid, waist circumference (WC), blood pressure, triglyceride level, and HDL can predict MS [4]. Furthermore, anthropometric measures like body mass index (BMI), WC, and waist-to-height ratio (WhtR) are often used as early markers of MS. Nevertheless, these metrics are unable to differentiate between subcutaneous fat and visceral obesity, and do not show an optimal predictive value [5,6]. So, more sophisticated metrics known as the cardiometabolic index (CMI) and lipid accumulation product (LAP) were suggested as better predictors of MS [7,8].

LAP is determined by the following equation for women: (WC [cm] − 58) × (triglycerides (TG) [mmol/L]); and for men: [WC (cm) − 65] × TG (mmol/L) [9]. As it includes two of the five components, it is a reliable predictor of MS. In individuals of different ethnicities (i.e., Afro-Caribbean), regardless of weight, LAP has been proposed to be an early marker of metabolic dysfunction and appears to have more clinical usefulness than BMI in predicting metabolic diseases, including MS and type 2 diabetes mellitus (T2DM) [7]. Moreover, LAP is linked to non-alcoholic fatty liver disease (NAFLD) and arterial stiffness and is thought to be a therapeutically valuable diagnostic tool for estimating insulin resistance and cardiometabolic risk [10,11]. On the other hand, CMI has been created as an additional and more refined biomarker because it accounts for blood lipids and obesity. It is specifically determined by multiplying the triglyceride/high-density lipoprotein ratio by the WhtR. In order to determine a possible risk of metabolic abnormalities, such as the existence of MS and/or T2DM, this parameter takes into account both the accumulation of abdominal fat and the presence of dyslipidemia. While these two indicators have been extensively studied and validated in the general population, data on athletes remain scarce. Consequently, there is a gap in the literature regarding the potential role of LAP and CMI in athletes and their accuracy in predicting MS in healthy subjects. Therefore, the aim of our study was to determine, in a large cohort of Olympic athletes, the prevalence of MS and its risk factors and the likelihood and accuracy of LAP and CMI of predicting athletes at high-risk for MS.

## 2. Materials and Methods

The Institute of Sports Medicine and Science in Rome is the division of the Italian Olympic Committee responsible for the medical evaluation of the elite athletes selected for participation in the Olympic Games. In our study, we included a cohort of 793 Olympic athletes evaluated during pre-participation screening for the 2012–2022 Olympic Games. Body height and weight were obtained in each subject, and body mass index (BMI) was calculated as weight (kg)/height (m)^2^. The WC was measured at the narrowest part between the lower border of the rib cage and the top of iliac crest to 0.1 cm with a tape measure while exhaling, with their feet set from 25 to 30 cm apart to distribute weight.

Body composition and fat mass percentage measurement was made with a Bioelectric Impedance Analysis (BIA 101 Quantum, Akern, Florence, Italy), with constant sinusoidal current, at an intensity of between 50 kHz and 400 μA. A blood test sample was collected, and the following biochemical indices were analyzed: total cholesterol, low-density lipoprotein cholesterol (LDL), HDL-cholesterol, triglycerides, and glycemia. Blood samples were collected in the institute of Sports Medicine and Science laboratory early in the morning and after at least 10 h fasting, and were analyzed on the same day. All of the blood tests were collected and analyzed in the same laboratory. Blood pressure was recorded in the sitting position before exercise testing, as recommended by the European Society of Cardiology guidelines [12].

MS was defined as meeting 3 or more of the 5 diagnostic criteria for MS that are defined according to the Adult Treatment Panel III (ATP III) report of the National Cholesterol Education Program (NCEP) [13]: fasting blood glucose ≥ 100 mg/dL or taking diabetes medications; TG ≥ 150 mg/dL; HDL-C < 40 mg/dL for men or <50 mg/dL for women; systolic blood pressure ≥ 130 mmHg or diastolic blood pressure ≥ 85 mmHg; and WC ≥ 90 cm for men or ≥80 cm for women [8,14].

Athletes who had 2 of the 5 diagnostic criteria and were arbitrarily defined as “high risk” for MS, while who had 1 of the 5 diagnostic criteria were defined as “low risk”. Those who had no criteria were subsequently defined as having “no risk”.

The LAP was calculated as follows for men: (WC − 65) × TG; for women: (WC − 58) × TG [8]. The cardiometabolic index (CMI) was calculated as the product of the triglycerides to high-density lipoprotein cholesterol (TG/HDL-C) and WHtR to predict cardiometabolic risk (CMR) in adults [15]. 

Athletes were engaged in a wide spectrum of sport disciplines, classified into four groups, as previously described [16]:

(1) Skill (technical disciplines): archery, golf, shooting, figure skating, sailing, curling, diving, and equestrian sports.
(2) Power (strength disciplines): weightlifting, Greco-Roman wrestling, judo, javelin, bobsleigh, skeleton, snowboard, swimming (<800 mt), alpine skiing, athletics (sprinting, shot put, and discus), and luge.
(3) Mixed discipline (alternate dynamic and strength components): soccer, volleyball, basketball, tennis, fencing, water polo, rhythmic gymnastics, taekwondo, badminton, beach volleyball, and softball.
(4) Endurance (primarily dynamic components): cycling, rowing, canoeing, triathlon, long-distance running, long-distance swimming (>800 mt), cross-country skiing, pentathlon, biathlon, speed skating, and Nordic combined.

CV risk factors were defined as follows:

(1) Family history for cardiovascular disease: Fatal or non-fatal CV events or/and established diagnosis of CV disease in first-degree male relatives aged under 55 years, or female relatives aged under 65 years [12], or evidence of carotid/peripheral atherosclerotic disease in first-degree relatives.
(2) Cigarette smoking: Defined as regular smokers of at least one cigarette per day.
(3) Overweight: BMI over 25; obesity: BMI over 30.

Each athlete of our cohort underwent a comprehensive pre-participation screening, including echocardiography, baseline ECG, and a stress test, which ruled out any significant cardiovascular diseases. This standardized screening ensures a healthy baseline within our cohort, allowing us to focus on relevant metabolic and performance metrics without the confounding effects of pre-existing cardiovascular conditions.

All of the athletes in our cohort trained more than 10 h per week.

The study design of the present investigation was evaluated and approved by the Review Board of the Institute of Medicine and Sports Science. All of the athletes included in this study were fully informed of the types and nature of the evaluation and signed a consent form, according to the Italian Law and Institute’s policy. All of the clinical data collected from the study population are stored in an institutional database. The work described was performed in accordance with The Code of Ethics of the World Medical Association (Declaration of Helsinki).

### Statistical Analysis

Categorical variables were expressed as frequencies and percentages, and were compared using Fisher’s exact test or a Chi-square test, as appropriate. Normality criteria were checked for any continuous variables, which were presented as mean and standard deviation (SD) and compared using Student’s *t*-test for independent data if they were normally distributed. Pearson’s correlation coefficient was used for the correlation analysis. The comparative analysis between athletes with different numbers of criteria for MS was performed using the Dunn test and Pairwise comparison method. The tables show the pooled *p*-value [of the comparison test with the 4 categories: MS (with MS), HR (high risk), LR (low risk), and NR (no risk)]. If pooled *p* < 0.05, a pairwise test was performed. All pairwise tests were considered significant if *p* < 0.05. To confirm the ability of LAP and CMI to accurately discriminate the “likely MS” phenotypes, an area under the curve (AUC) was obtained using a receiver operating characteristic curve (ROC curve). The optimal cut-off value, sensitivity, specificity, and Youden Index of the LAP and CMI were obtained through the ROC curve. Moreover, the odds ratio and 95% confidence interval were obtained through logistic regression after adjusting for age, sex, fat mass, TC, LDL, and BMI. Statistical analysis was performed with STATA Statistics for Windows (SE, version 17) software.

## 3. Results

We enrolled 793 Olympic athletes, 433 males (54.6%), mean age 24.8 ± 5 years, mean body weight 73.6 ± 14.7 kg, and mean BMI 23.2 ± 3.1 kg/m^2^. Athletes were divided according to sporting disciplines as follows: skill (90, 11.3%), power (246, 31%), mixed discipline (340, 42.9%), and endurance (117, 14.7%). Most of the athletes were Caucasians (755, 95.2%). In total, 62 athletes (7.8%) were smokers, 142 (17.9%) had familiarity with cardiovascular disease, and 82 (10.3%) had familiarity with dyslipidemia.

The main differences between athletes with an increasing number of criteria for MS are listed in Table 1. In total, 6 athletes (0.8%) reached the criteria for MS (at least three criteria over 5), 72 athletes (9.1%) had two criteria and were defined as “high risk” for MS, 300 athletes has one criteria (37.8%) defined as “low risk”, and 415 athletes (52.3%) had no criteria and were subsequently defined as “no risk”. LAP presented progressively increasing values starting from 11.3 ± 7.8 in those at no risk, 37.1 ± 26.6 in those at low risk, 54.7 ± 45.4 for athletes at high risk, and 109.4 ± 57.6 in athletes with MS (*p* < 0.0001). The same numerical behavior was observed for CMI, with 0.45 ± 0.22 for those at no risk, 0.76 ± 0.46 for those with low risk, 1.21 ± 0.87 for those with high risk, and 4.23 ± 4.42 for those with MS (*p* < 0.0001).

In athletes with at least two criteria (HR and MS), a higher prevalence of male athletes was registered (respectively, 100% and 73.6%) compared to athletes with one or no criteria (respectively, 49% and 54.7%, *p* = 0.0002). Significant differences in anthropometric parameters were highlighted in those with HR and MS, with higher body weight (*p* < 0.0001), higher BMI and BSA (*p* < 0.0001), and higher prevalence of overweight athletes (*p* < 0.0001). The lipid profile comparison showed progressively lower concentrations of TC, LDL, and TG and higher HDL-cholesterol values from MS athletes to athletes with no risk for MS (all *p* < 0.0001). The same results were observed also for SBP, DBP, and glycemia (all *p* < 0.0001). The type of sport did not show a relationship with the number of criteria for MS.

In fact, in power and mixed athletes, similar prevalence values were found (respectively, 0.382 and 0.123). In skill athletes, only between NR and LW was a significant difference found (*p* = 0.012). In athletes practicing endurance disciplines, a lower number of criteria were observed (*p* = 0.0006), with a progressive prevalence reduction from 19.5% in those with no criteria to 10.7% in those with one criteria and 5.5% with two criteria. No athletes with MS practiced endurance disciplines.

Finally, we have grouped athletes with MS and those with high risk in “likely MS” (*n* = 78, 9.8%) and athletes with low and no risk in “unlikely MS” (*n* = 715, 90.2%). The usefulness of the LAP and CMI for identifying athletes with likely MS through ROC curves is presented in Table 2. For the LAP, the AUC was 0.80 (95% CI, 0.77–0.83) for all of the participants, 0.81 (95% CI, 0.77–0.84) for male athletes, and 0.77 (95% CI, 0.72–0.81) for female athletes. For the CMI, the AUC was 0.79 (95% CI, 0.76–0.82) for all of the participants, 0.78 (95% CI, 0.74–0.82) for male athletes, and 0.74 (95% CI, 0.69–0.78) for female athletes, thereby indicating good discrimination ability. In addition, the optimal cut-off values for identifying the likely MS group were, respectively, 34.66 for LAP and 0.776 for CMI (general population). Different gender cut-offs were identified with a LAP of 38.79 for men and 14.16 for women and a CMI of 0.881 for men and 0.965 for women. The adjusted OR (95% CI) of the LAP and CMI for the “likely MS” athletes is presented in Table 3. In the overall population, after controlling for age, sex, fat mass, TC, LDL, and BMI, the LAP ≥ 34.66 had a hazard ratio of 7.22 [3.75–13.89], *p* < 0.0001, and the CMI ≥ 0.776 had a hazard ratio of 5.37 [2.96–9.73], *p* < 0.0001. For males, after adjusting for age, fat mass, TC, LDL, and BMI, the LAP ≥ 38.79 had a hazard ratio of 6.22 [2.92–13.24], *p* < 0.0001, and CMI ≥ 0.881 had a hazard ratio of 4.80 [2.42–9.50], *p* < 0.0001. On the other hand, for female athletes, LAP ≥ 14.16 and CMI ≥ 0.965 had, respectively, a hazard ratio of 29.70 [3.50–252.06], *p* = 0.002, and 97.18 [16.00–590.37], *p* < 0.0001.

In Table 4 are presented the proposed gender specific cut-offs for both LAP and CMI for identifying athletes with MS or those that will likely develop MS.

## 4. Discussion

Currently, MS is considered one of the major public health issues [17]. Indeed, MS has been related to a 1.5-fold increase in the risk of all-cause death and a 2-fold increase in the risk of CVD mortality and stroke [4].

A large portion of the population encounters undiagnosed MS, despite the prevalence of MS being significant [8,18,19]. Indeed, accurate diagnosis and treatment of MS are crucial for global- and individual-level prevention efforts, since the disease is becoming more common in all age groups. Moreover, primary prevention is essential for lowering the burden and expense of healthcare-related illnesses in the future [2]. In this setting, regular moderate physical activity (PA) improves blood pressure, body composition, lipid profile, and insulin sensitivity. Conversely, one of the main risk factors for MS and overall mortality is poor levels of physical fitness [20,21]. Research indicates that fulfilling or exceeding PA guidelines has a negative correlation with the likelihood of developing MS and enhances parameters in those who already have MS or are at risk [22]. In addition, patients who no longer fit MS criteria may be reclassified as a result of an exercise program that improves any of the MS markers. About 30.5% of healthy people enrolled in the study by Katzmarzyk et al. were reclassified as no longer having MS due to the combined effect on the improvement of MS indicators following 20 weeks of supervised exercise [23].

Different anthropometric measures like body mass index (BMI), WC, and WHtR have been proposed for predicting MS. However, they present different limits, and new index parameters such as LAP and CMI have recently shown promising results [5,6,7,8,9,10,11,12]. Currently, WC and the concentration of TG at fasting are combined to form LAP, a measure of visceral obesity. Anatomical and biochemical alterations associated with lipid overaccumulation in humans can be described by using anthropometric and biochemical data to compute LAP simultaneously [8,24]. LAP is frequently utilized as a predictor of CVD and as a marker of metabolic disorders. It is a clinical indicator of visceral obesity and has been suggested as an easy, affordable, and reliable way to calculate cardiovascular risk and death because the gold-standard techniques for assessing visceral fat are costly and the measurement of WC cannot differentiate between visceral and subcutaneous fat [25,26]. According to Rotter et al., people with T2DM, obesity, and MS had far greater LAP values than people without these disorders [25]. Additionally, these authors discovered a negative link with HDL-cholesterol and a positive and substantial correlation with insulin, glycemia, and total cholesterol [25]. Moreover, Guo et al. investigated the relationship between LPA and metabolic parameters and discovered that this index is a helpful indicator for MS diagnosis and screening [26]. However, the LAP of men and women cannot easily be compared, since different WC corrections are employed. Compared to WC and BMI, WHtR has been demonstrated to be a more accurate predictor of coronary heart disease and cardiovascular risk factors. Consequently, the product of the TG/HDL-C ratio and WHtR has led to the proposal of a new parameter, the CMI. Furthermore, CMI has been demonstrated to be a key factor in MS and a strong predictor of CVD [7]. With a cut-off of 0.84, the CMI is the most practical and trustworthy index to be used in clinical practice for identifying MS in obese women [27]. However, these indexes have not been studied in a healthy population practicing physical activity, such as athletes.

In our study, we enrolled 794 Olympic athletes, practicing different sports, who were mostly (90.1%) at low or no risk for MS. Remarkably, only a small subset of individuals (9.9%) showed MS or high risk for MS. This confirms that physical activity is associated with lower prevalence of MS [20]. Indeed, regular–moderate PA contributes to improving insulin sensitivity, lipid profile, blood pressure, and body composition [20,21]. However, some athletes may present higher blood pressure values, cholesterol levels, and glycemia levels, being exposed to higher risk for developing MS, as in our cohort. Indeed, athletes are not immune to MS onset and, consequently, to a higher risk for CAD [28]. Thus, individuating patients at risk is crucial to prevent MS development and reduce the risk of adverse outcomes.

From a gender-specific perspective, males are more likely than females to have MS, while females who have MS are more likely to develop CVD. Males are said to be more at risk of T2DM and hypertension among the MS components, whereas females are more likely to have dyslipidemia and abdominal obesity [29]. In our study, sex had also an impact when in comes to MS risk classifying of athletes. Indeed, all of the individuals at HR and more than 70% of those with MS were males. However, the prevalence of MS varies depending on the population examined, the definition employed, and the age, ethnicity, and sex of the affected person. A person’s diet, degree of physical activity, genetic background, and degree of over- or under-nutrition all have an impact on how common the condition and its components are. Age-related increases in the prevalence of MS are particularly noticeable during the pre-menopausal to post-menopausal transition [30]. However, in our study, all of the athletes were young. Thus, generalized statements regarding sex differences in prevalence may be misleading due to the potential influence of these numerous confounding factors.

On the other hand, athletes with increased BMI, BSA, or overweight were more frequently individuals satisfying the criteria for HR MS or MS. Thus, preventing measures should be applicated in the early stage or before developing MS. Methods that can effectively reduce body fat include changing diet and modifying energy expenditure through exercise, especially visceral fat.

Moreover, the prevalence of most sporting categories did not significantly change according to the number of criteria. In fact, in power, mixed, and skill athletes, there were no significant differences between those at HR and LW or NR. However, endurance athletes were more frequently individuals with NR or LW than HR (19.5%, 10.7%, and 5.5%, respectively). Indeed, it seems that endurance training has a more favorable effect on glucose and insulin homeostasis and lipid profile than strength training and, consequently, on MS [31].

However, the primary strength of this study is the assessment of LAP and CMI in identifying patients at risk for MS. Indeed, athletes at HR or with MS presented significantly higher values of LAP and CMI than those LR or NR, as illustrated in Table 1. In the sub-analysis, patients were classified into those ‘’likely’’ to develop MS, if there were at least two criteria, and ‘’unlikely’’ if there were no or one criteria for MS. Indeed, LAP and CMI confirmed to be good predictors of patients at risk for MS, with good accuracy in both male and female athletes (AUCs of 0.80 and 0.79, respectively). This confirms the good accuracy of both markers in predicting MS already studied in the general population, especially individuals with obesity [32,33,34].

Individuating athletes at risk would help focus on the frequent monitoring of MS components, the further evaluations of early screenings for MS-related diseases, and mitigating the modifiable underlying risk factors through lifestyle and physical activity changes [35]. For that purpose, we individuated the ideal cut-off, corrected for potential confounders, for identifying athletes likely to develop MS. Indeed, in the general population, LAP ≥ 34.66 and CMI ≥ 0.776 conferred a 7-fold and 5-fold risk for developing MS, respectively. However, these values had different adjusted cut-offs between males and females. Our study individuated LAP cut-offs as between 38.79 and 14.16 and CMI cut-offs as between 0.881 and 0.965 for male and female athletes, respectively. These cut-offs are slightly different from those described in other studies. Because adipose tissue accumulates around the hips and thighs in women and around the trunk and lower abdominal part in men, physiological variances in height, weight, and body composition between the sexes may be the cause of the disparities in LAP measures between the performance of males and females [36]. According to Haijiang Dai et al. [37], MS prevalence rose in both the male and female groups when LAP levels increased. For men and women, respectively, LAPs ≥ 30.5 and 23.0 were shown to be the maximum values for sensitivity and specificity in the diagnosis of MS. On the contrary, the highest sensitivity and specificity were found with LAP cut-off values of 34.2 for the entire sample in a study by Nascimento-Ferreira et al. [5]. Following age stratification, the LAP cut-off values for males and females under 50 were 64.1 and 38, respectively, while for subjects over 50, the values were 36.4 and 34.2 for males and females, respectively. On the other hand, Lazzer et al. individuated 0.84 as a CMI cut-off for individuating women with obesity at risk for developing MS, while male cut-offs for CMI seem to be lower [15,27,38]. As young athletes generally present lower WC and triglyceride levels, this could explain the lower LAP and CMI values in our study compared to those reported in the other studies. Nevertheless, this study highlights the good accuracy of both indexes in predicting MS risk and their usefulness in the early diagnosis of MS among athletes.

LAP and CMI may be routinely calculated for all athletes to identify those at risk for metabolic syndrome. This would allow for the early detection of potential cardiometabolic issues and enable clinicians to place “high risk” athletes under more intensive follow-up and monitoring. By incorporating these indices into regular health assessments, we could ensure that athletes who may be predisposed to metabolic syndrome receive timely interventions, promoting better long-term health outcomes and reducing the risk of developing more severe conditions.

## 5. Limitations

Our study presents several limitations. Firstly, it is a retrospective observational study. Therefore, future prospective studies are needed to verify the suggested instruments and assess their predictive significance for athletes’ cardiovascular health on a large scale. The population’s demographics, which included a narrow age range (most of them had median age of 25), a sizable sample size, and were restricted to a single center, indicate another limit. Furthermore, this restricts generalizability because this study primarily consists of athletes of Italian nationality and Caucasian ethnicity. Lastly, a variety of factors, including food and prolonged periods of hard training, can affect body composition. Olympic athletes were assessed by our research at all stages throughout the year, including peak training, competitions, and post-training periods like holidays. Furthermore, our indexes’ therapeutic efficacy has not yet been established, but they do represent an effort to develop models and scores that will help identify athletes who may be at risk for MS. Large-scale randomized trials are nevertheless required.

## 6. Conclusions

Athletes have lower prevalence of MS compared to the general population. However, they are not immune to developing MS and related issues, including CVD. Therefore, using prediction models, including LAP and CMI, aids in the individuation of athletes at different risks for MS. Indeed, the ROC curve analyses of LAP and CMI showed good diagnostic accuracy as a supplementary screening tool in predicting athletes at high risk for MS, despite the low prevalence of MS in our cohort. The early detection of individuals at risk for MS may contribute to establishing an early prevention strategy, including physical, nutraceutical, or pharmacological management. Randomized prospective studies are necessary to validate their role on a larger scale and their utility in everyday clinical practice. New trials are required to validate the role of LAP and CMI in predicting cardiovascular events and mortality among athletes at risk.

## Figures and Tables

**Table 1:** Differences in main clinical and anthropometric parameters, blood test results, and indexes between athletes with different numbers of criteria for metabolic syndrome.

*N* = 793	MS	High Risk	Low Risk	No Risk	*p* Pooled	*p* Pairwise
	Three criteria	Two criteria	One criteria	No criteria		
*N*, (%)	6 (0.8)	72 (9.1)	300 (37.8)	415 (52.3)		
LAP	109.4 ± 57.6	54.7 ± 45.4	37.1 ± 26.6	11.3 ± 7.8	<0.0001	MS vs. HR, *p* = 0.007; MS vs. LR, *p* < 0.0001; MS vs. NR, *p* < 0.0001; HR vs. LR, *p* < 0.0001; HR vs. NR, *p* < 0.0001; LR vs. NR, *p* < 0.0001
CMI	4.23 ± 4.42	1.21 ± 0.87	0.76 ± 0.46	0.45 ± 0.22	<0.0001	MS vs. HR, *p* < 0.0001; MS vs. LR, *p* < 0.0001; MS vs. NR, *p* < 0.0001; HR vs. LR, *p* < 0.0001; HR vs. NR, *p* < 0.0001; LR vs. NR, *p* < 0.0001
Male, *n* (%)	6 (100)	53 (73.6)	147 (49)	227 (54.7)	0.0002	MS vs. LR, *p* = 0.013; MS vs. NR, *p* = 0.026; HR vs. LR, *p* = 0.0002; HR vs. NR, *p* = 0.002; LR vs. NR, *p* = 0.132; MS vs. HR, *p* = 0.151.
Age, years	26.7 ± 4.7	25.4 ± 6	24.4 ± 5	24.9 ± 4.8	0.303	-
Weight, kg	106.5 ± 20.6	81.9 ± 16.9	72.7 ± 13.8	72.3 ± 13.8	<0.0001	MS vs. HR, *p* = 0.001; MS vs. LR, *p* < 0.0001; MS vs. NR, *p* < 0.0001; HR vs. LR, *p* < 0.0001; HR vs. NR, *p* < 0.0001; LR vs. NR, *p* = 0.696.
BMI, kg/m^2^	29.3 ± 5.4	25.2 ± 4.1	23.2 ± 2.9	22.7 ± 2.6	<0.0001	MS vs. HR, *p* = 0.027; MS vs. LR, *p* < 0.0001; MS vs. NR, *p* < 0.0001; HR vs. LR, *p* < 0.0001; HR vs. NR, *p* < 0.0001; LR vs. NR, *p* = 0.006
BMI > 25 kg/m^2^	5 (83.3)	29 (40.3)	76 (25.3)	75 (18.1)	<0.0001	MS vs. HR, *p* = 0.041; MS vs. LR, *p* = 0.001; MS vs. NR, *p* < 0.0001; HR vs. LR, *p* < 0.0001; HR vs. NR, *p* < 0.0001; LR vs. NR, *p* = 0.018.
BSA	2.35 ± 0.26	2 ± 0.24	1.87 ± 0.23	1.87 ± 0.24	<0.0001	MS vs. HR, *p* = 0.001; MS vs. LR, *p* < 0.0001; MS vs. NR, *p* < 0.0001; HR vs. LR, *p* < 0.0001; HR vs. NR, *p* < 0.0001; LR vs. NR, *p* = 0.945.
Fat mass, %	17.1 ± 7.8	16.5 ± 8.1	16.6 ± 7.2	14.4 ± 6.2	0.0004	HR vs. NR, *p* = 0.018; LR vs. NR, *p* < 0.0001; MS vs. HR, *p* = 0.877; MS vs. LR, *p* = 0.866; MS vs. NR, *p* = 0.300; HR vs. LR, *p* = 0.973;
WC, cm	121.7 ± 22	111.6 ± 23.9	105.4 ± 24.3	77.5 ± 9.8	<0.0001	MS vs. NR, *p* < 0.0001; HR vs. NR, *p* < 0.0001; LR vs. NR, *p* < 0.0001; MS vs. HR, *p* = 0.326; MS vs. LR, *p* = 0.107; HR vs. LR, *p* = 0.056.
Afro-Caribbean, *n* (%)	0 (0)	0 (0)	13 (4.3)	25 (6)	0.142	-
Smokers, *n* (%)	1 (16.7)	12 (16.7)	26 (8.7)	23 (5.5)	0.006	HR vs. LR, *p* = 0.037; HR vs. NR, *p* = 0.0005; MS vs. HR, *p* = 0.976; MS vs. LR, *p* = 0.500; MS vs. NR, *p* = 0.245; LR vs. NR, *p* = 0.099.
Family history for CVD, *n* (%)	0 (0)	11 (15.3)	47 (15.7)	84 (20.2)	0.258	-
Training hours/week	23 ± 2.3	22.3 ± 9	22.3 ± 9.3	24 ± 10.2	0.310	-
TC, mg/dL	184.8 ± 24	172.3 ± 8.1	175.7 ± 27.4	161 ± 27.7	<0.0001	MS vs. NR, *p* = 0.036; HR vs. NR, *p* = 0.001; LR vs. NR, *p* < 0.0001; MS vs. HR, *p* = 0.209; MS vs. LR, *p* = 0.421; HR vs. LR, *p* = 0.320;
LDL, mg/dL	97 ± 35	98.5 ± 21.9	97.8 ± 22.4	84.3 ± 22.7	<0.0001	HR vs. NR, *p* < 0.0001; LR vs. NR, *p* < 0.0001; MS vs. HR, *p* = 0.877; MS vs. LR, *p* = 0.931; MS vs. NR, *p* = 0.180; HR vs. LR, *p* = 0.804;
HDL, mg/dL	42 ± 15	54 ± 13	63 ± 15	64.7 ± 14	<0.0001	MS vs. HR, *p* = 0.037; MS vs. LR, *p* = 0.0006; MS vs. NR, *p* < 0.0001; HR vs. LR, *p* < 0.0001; HR vs. NR, *p* < 0.0001; LR vs. NR, *p* = 0.245.
TG, mg/dL	228.8 ± 184	94.5 ± 50.1	74.3 ± 35.7	62.9 ± 22.1	<0.0001	MS vs. HR, *p* < 0.0001; MS vs. LR, *p* < 0.0001; MS vs. NR, *p* < 0.0001; HR vs. LR, *p* < 0.0001; HR vs. NR, *p* < 0.0001; LR vs. NR, *p* < 0.0001
SBP, mmHg	129.2 ± 6.1	121.7 ± 10.9	110.2 ± 11.5	108.7 ± 9.9	<0.0001	MS vs. LR, *p* < 0.0001; MS vs. NR, *p* < 0.0001; HR vs. LR, *p* < 0.0001; HR vs. NR, *p* < 0.0001; LR vs. NR, *p* = 0.064; MS vs. HR, *p* = 0.104.
DBP, mmHg	81.7 ± 6.2	74.9 ± 8	68.8 ± 7.6	67.3 ± 7.1	<0.0001	MS vs. LR, *p* < 0.0001; MS vs. NR, *p* < 0.0001; HR vs. LR, *p* < 0.0001; HR vs. NR, *p* < 0.0001; LR vs. NR, *p* = 0.005; MS vs. HR, *p* = 0.052;
Glycemia, mg/dL	104.3 ± 21.5	95.6 ± 7.1	90.8 ± 8.9	88.3 ± 5.5	<0.0001	MS vs. HR, *p* = 0.027; MS vs. LR, *p* = 0.0005; MS vs. NR, *p* < 0.0001; HR vs. LR, *p* < 0.0001; HR vs. NR, *p* < 0.0001; LR vs. NR, *p* < 0.0001.
WC ≥ 88 cm female, *n* (%)	0 (0)	17 (23.6)	119 (39.7)	0 (0)	<0.0001	MS vs. LR, *p* = 0.048; HR vs. LR, *p* = 0.010; HR vs. NR, *p* < 0.0001; LR vs. NR, *p* < 0.0001; MS vs. HR, *p* = 0.182.
WC ≥ 102 cm, male, *n* (%)	5 (83.3)	37 (51.4)	86 (28.7)	0 (0)	<0.0001	MS vs. LR, *p* = 0.003; MS vs. NR, *p* < 0.0001; HR vs. LR, *p* < 0.0001; HR vs. NR, *p* < 0.0001; LR vs. NR, *p* < 0.0001; MS vs. HR, *p* = 0.135;
HDL < 40 mg/dL, male, *n* (%)	4 (66.6)	7 (9.7)	8 (2.7)	0 (0)	<0.0001	MS vs. HR, *p* < 0.0001; MS vs. LR, *p* < 0.0001; MS vs. NR, *p* < 0.0001; HR vs. LR, *p* = 0.006; HR vs. NR, *p* < 0.0001; LR vs. NR, *p* = 0.0008.
HDL < 50 mg/dL, female, *n* (%)	0 (0)	8 (11.1)	15 (5)	0 (0)	<0.0001	HR vs. NR, *p* < 0.0001; LR vs. NR, *p* < 0.0001; MS vs. HR, *p* = 0.395; MS vs. LR, *p* = 0.575; HR vs. LR, *p* = 0.053;
SBP ≥ 130 mmHg, *n* (%)	4 (66.6)	33 (45.8)	28 (9.3)	0 (0)	<0.0001	MS vs. LR, *p* < 0.0001; MS vs. NR, *p* < 0.0001; HR vs. LR, *p* < 0.0001; HR vs. NR, *p* < 0.0001; LR vs. NR, *p* < 0.0001; MS vs. HR, *p* = 0.332.
DBP ≥ 85 mmHg, *n* (%)	0 (0)	7 (9.7)	2 (0.7)	0 (0)	<0.0001	HR vs. LR, *p* < 0.0001; HR vs. NR, *p* < 0.0001; MS vs. HR, *p* = 0.430; MS vs. LR, *p* = 0.841; LR vs. NR, *p* = 0.096.
TG ≥ 150 mg/dL, *n* (%)	3 (50)	12 (16.7)	6 (2)	0 (0)	<0.0001	MS vs. HR, *p* = 0.043; MS vs. LR, *p* < 0.0001; MS vs. NR, *p* < 0.0001; HR vs. LR, *p* < 0.0001; HR vs. NR, *p* < 0.0001; LR vs. NR, *p* = 0.003.
GI, *n* (%)	4 (66.6)	23 (31.9)	36 (12)	0 (0)	<0.0001	MS vs. LR, *p* < 0.0001; MS vs. NR, *p* < 0.0001; HR vs. LR, *p* < 0.0001; HR vs. NR, *p* < 0.0001; LR vs. NR, *p* < 0.0001; MS vs. HR, *p* = 0.088.
Rest HR, bpm	85 ± 16.6	73 ± 13	70.3 ± 7.6	69.4 ± 13	0.009	MS vs. HR, *p* = 0.038; MS vs. LR, *p* = 0.013; MS vs. NR, *p* = 0.004; HR vs. NR, *p* = 0.032; HR vs. LR, *p* = 0.148; LR vs. NR, *p* = 0.379.
Power, *n* (%)	3 (50)	22 (30.5)	84 (28)	137 (33)	0.382	-
Skill, *n* (%)	1 (16.7)	11 (15.3)	43 (14.3)	35 (8.4)	0.059	LR vs. NR, *p* = 0.012; MS vs. HR, *p* = 0.929; MS vs. LR, *p* = 0.872; MS vs. NR, *p* = 0.475; HR vs. LR, *p* = 0.838; HR vs. NR, *p* = 0.067;
Endurance, *n* (%)	0 (0)	4 (5.5)	32 (10.7)	81 (19.5)	0.0006	HR vs. NR, *p* = 0.003; LR vs. NR, *p* = 0.001; MS vs. HR, *p* = 0.559; MS vs. LR, *p* = 0.399; MS vs. NR, *p* = 0.229; HR vs. LR, *p* = 0.188.
Mixed, *n* (%)	2 (33.3)	35 (48.6)	141 (47)	162 (39)	0.123	-

Abbreviations: BMI: body mass index; BSA: body surface area; CMI: cardiometabolic index; CVD: cardiovascular diseases; DBP: diastolic blood pressure; GI: glucose intolerance; HDL: high-density lipoprotein; HR: heart rate; LAP: lipid accumulation product; LDL: low-density lipoprotein; MS: metabolic syndrome; SBP: systolic blood pressure; TC: total cholesterol; TG: triglycerides; WC: waist circumference.

**Table 2:** Areas under the receiver operating characteristics curve (AUC) for detecting athletes with likely Metabolic Syndrome with LAP and CMI. Abbreviations: AUC: area under the curve; CMI: cardiometabolic index; LAP: lipid accumulation product.

	AUC (Continuous Variable)	Cut-Off (Youden)	Sensitivity	Specificity	AUC (Cut-Off)	Youden Index
LAP	0.80 (0.77–0.83)	34.66	67%	80%	0.73	0.468
CMI	0.79 (0.76–0.82)	0.776	68%	79%	0.74	0.474
Males, *n* = 433						
LAP	0.81 (0.77–0.84)	38.79	68%	82%	0.75	0.499
CMI	0.78 (0.74–0.82)	0.881	66%	79%	0.72	0.450
Females, *n* = 360						
LAP	0.77 (0.72–0.81)	14.16	95%	52%	0.73	0.466
CMI	0.74 (0.69–0.78)	0.965	53%	94%	0.73	0.465

**Table 3:** OR and 95% CI of cut-offs for the identification of athletes with “likely Metabolic Syndrome” associated with LAP and CMI. Abbreviations: BMI: body mass index; CI: confidence intervals; CMI: cardiometabolic index; H: high risk; L: low risk; LAP: lipid accumulation product; LDL: low-density lipoprotein; MS: metabolic syndrome; No: no risk; OR: odds ratio; TC: total cholesterol.

	Unadjusted	Adjusted ^1^		Unadjusted ^2^			Adjusted ^1,2^	
Cut-Off	“Likely MS” (MS + H vs. L + No)	“Likely MS” (MS + H vs. L + No)	Low	High	MS	Low	High	MS
All athletes								
LAP ≥ 34.66	8.07 [4.87–13.38] *p* < 0.001	7.22 [3.75–13.89] *p* < 0.001	6.07 [3.86–12.37] *p* < 0.001	57.06 [26.32–123.72] *p* < 0.001	>1000 *p* < 0.001	6.73 [2.98–13.99] *p* < 0.001	48.60 [21.69–108.89] *p* < 0.001	>1000 *p* < 0.001
CMI ≥ 0.776	8.19 [4.92–13.62] *p* < 0.001	5.37 [2.96–9.73] *p* < 0.001	6.06 [3.42–10.00] *p* < 0.001	8.19 [4.92–13.63] *p* < 0.001	15.12 [1.76–130.72] *p* = 0.013	4.01 [1.30–8.93] *p* < 0.001	6.30 [4.08–9.74] *p* < 0.001	4.84 [0.44–0.52] *p* = 0.195
Sex: male								
LAP ≥ 38.79	9.64 [5.25–17.69] *p* < 0.001	6.22 [2.92–13.24] *p* < 0.001	7.64 [3.25–15.69] *p* < 0.001	55.74 [19.98–155.49] *p* < 0.001	>1000 *p* < 0.001	4.36 [1.95–11.67] *p* < 0.001	38.24 [13.09–111.71] *p* < 0.001	>1000 *p* < 0.001
CMI ≥ 0.881	7.16 [3.96–12.96] *p* < 0.001	4.80 [2.42–9.50] *p* < 0.001	5.16 [1.96–10.99] *p* < 0.001	7.74 [4.64–12.89] *p* < 0.001	13.72 [1.58–118.76] *p* = 0.017	2.72 [1.38–7.39] *p* < 0.001	5.30 [3.07–9.16] *p* < 0.001	4.33 [0.37–50.14] *p* = 0.240
Sex: female								
LAP ≥ 14.16	19.42 [2.56–147.15] *p* = 0.004	29.70 [3.50–252.06] *p* = 0.002	7.6 [4.25–13.23] *p* < 0.001	19.42 [2.56–147.15] *p* = 0.004	-	34.49 [17.18–69.27] *p* < 0.001	96.03 [8.87–1039.27] *p* < 0.001	-
CMI ≥ 0.965	16.93 [6.21–46.15] *p* < 0.001	97.18 [16.00–590.37] *p* < 0.001	4.21 [1.71–11.55] *p* < 0.001	16.93 [6.21–46.15] *p* < 0.001	-	21.75 [3.75–120.24] *p* < 0.001	83.01 [14.44–477.06] *p* < 0.001	-

^1^ Adjusted for age, sex (if not in sub-analysis), fat mass, TC, LDL, and BMI. ^2^ Reference variable: “no risk”.

**Table 4:** Sex-specific cut-offs proposed by the study. Abbreviations: CMI: cardiometabolic index; LAP: lipid accumulation product.

	Male	Female
LAP	≥38.79	≥14.16
CMI	≥0.881	≥0.965

## Data Availability

The original contributions presented in the study are included in the article, further inquiries can be directed to the corresponding author.
